# ErCas12a and T5exo-ErCas12a Mediate Simple and Efficient Genome Editing in Zebrafish

**DOI:** 10.3390/biology11030411

**Published:** 2022-03-08

**Authors:** Bingzhou Han, Yage Zhang, Yang Zhou, Biao Zhang, Christopher J. Krueger, Xuetong Bi, Zuoyan Zhu, Xiangjun Tong, Bo Zhang

**Affiliations:** 1 Key Laboratory of Cell Proliferation and Differentiation of the Ministry of Education, Peking University Genome Editing Research Center, College of Life Sciences, Peking University, Beijing 100871, China; bzhan@pku.edu.cn (B.H.); ygzhang@pku.edu.cn (Y.Z.); yangzhou@stu.pku.edu.cn (Y.Z.); 1800012157@pku.edu.cn (B.Z.); 1901110469@pku.edu.cn (X.B.); zyzhu@pku.edu.cn (Z.Z.); xiangjuntong@pku.edu.cn (X.T.); 2 Department of Biomedical Engineering, College of Engineering, Peking University, Beijing 100871, China; ckrueger3@gatech.edu; 3 Wallace H. Coulter Department of Biomedical Engineering, Georgia Institute of Technology and Emory University, Atlanta, GA 30332, USA

**Keywords:** ErCas12a, genome editing, knockin, MMEJ, NHEJ, zebrafish

## Abstract

**Simple Summary:**

CRISPR/Cas9 enables efficient mutagenesis and generation of various knockout and knockin alleles in many species including zebrafish. However, the application of the Cas12a nuclease in zebrafish is far from ideal due to demanding experimental conditions, especially the requirements for delivery such as a purified protein and the heatshock of embryos. Here we show that ErCas12a, the only Cas12a reported to be effective when injected as mRNA in zebrafish, is highly efficient for large fragment knockin via either microhomology-mediated or non-homologous end joining pathways with mild heatshock conditions. Moreover, we fused T5 exonuclease to ErCas12a and found that the fusion protein could efficiently induce gene knockout and knockin without heatshock. Therefore, we demonstrated the efficacy of multiple genome-editing applications using ErCas12a and its variant with simplified conditions in zebrafish.

**Abstract:**

In zebrafish, RNA-guided endonucleases such as Cas9 have enabled straightforward gene knockout and the construction of reporter lines or conditional alleles via targeted knockin strategies. However, the performance of another commonly used CRISPR system, Cas12a, is significantly limited due to both the requirement of delivery as purified protein and the necessity of heatshock of injected embryos. To explore the potential of CRISPR/Cas12a-mediated genome editing and simplify its application in zebrafish, we took advantage of the recently reported mRNA-active ErCas12a and investigated its efficacy for the knockin of large DNA fragments, such as fluorescent reporter genes. For knockin via either microhomology-mediated end joining (MMEJ) or non-homologous end joining (NHEJ) pathways, ErCas12a-injected embryos with a brief heatshock displayed comparable knockin efficiency with Cas9 injection. Through the fusion of T5 exonuclease (T5exo) to the N-terminus of ErCas12a (T5exo-ErCas12a), we further demonstrated high efficiency gene knockout and knockin at a normal incubation temperature, eliminating the embryo-damaging heatshock step. In summary, our results demonstrate the feasibility of ErCas12a- and T5exo-ErCas12a-mediated genome manipulation under simplified conditions, and further expand the genome editing toolbox for various applications in zebrafish.

## 1. Introduction

The CRISPR/Cas system, including the type II effector Cas9 and the type V effector Cas12a, has been widely adopted for genome editing both ex vivo and in vivo. To date, the most commonly used Cas12a (formerly known as Cpf1) nucleases were obtained from either *Acidaminococcus* or *Lachnospiraceae*, both of which recognize a thymine-rich protospacer adjacent motif (PAM) and cut DNA distal to the PAM in a staggered fashion leaving a sticky DNA double-strand break (DSB) end with a four-nucleotide 5′ overhang [1]. Given the fact that Cas9 recognizes a guanine-rich PAM and generates blunt DSB ends [2,3,4], the application of Cas12a may provide benefits, including expanded target site choice, such as T-rich introns, non-coding RNAs, and UTRs [1]; facile multiplexed genome editing enabled by the pre-crRNA processing ability of Cas12a [5,6,7,8,9]; and increased efficiency of homology-based knockin due to the sticky ends generated by Cas12a cutting [10,11].

In zebrafish (*Danio rerio*), however, while the use of *Streptococcus pyogenes* Cas9 nuclease (hereafter referred to as Cas9) is as convenient, efficient, and versatile as in ex vivo systems [12,13,14,15,16,17,18,19,20], the application of Cas12a is still limited [11,21,22]. Cas12a generally showed poor editing efficiency in zebrafish, and optimal mutagenesis required injection of AsCas12a/crRNA or LbCas12a/crRNA in the form of ribonucleoproteins (RNPs) followed by heatshock for four hours, which may damage embryos [21,22]. Recently, Cas12a identified from *Eubacterium rectale* (ErCas12a, also known as MAD7 nuclease), was reported to induce efficient mutagenesis when injected as mRNA in zebrafish embryos, but also requires heatshock treatment [11]. Interestingly, the integration of large DNA fragments into the zebrafish genome has been frequently reported with the use of Cas9 in the form of mRNA [12,14,16,17,20,23,24], while, to date, no studies have reported successful NEHJ- or MMEJ-mediated knockin in zebrafish using the highly efficient RNP form of Cas9 [25,26,27]. Therefore, we hypothesized that the mRNA-active ErCas12a could be an ideal tool for large fragment knockin in zebrafish, particularly for homology-based approaches, which might be facilitated by the staggered DSBs. In addition, its different target site selection criteria compared with Cas9 should greatly increase the targetable range as well as flexibility for the experimental design to achieve large fragment integration, especially for T-rich sequences such as introns.

Here we report efficient and heritable microhomology-mediated end joining (MMEJ) and non-homologous end joining (NHEJ)-based large fragment integration mediated by *ErCas12a* mRNA in zebrafish. Using a limited heatshock process, *ErCas12a* induced efficient fluorescent reporter expression from donor plasmid knockin, comparable with *Cas9* mRNA, in multiple loci. Moreover, we further bypassed the heatshock process in Cas12a application by fusing T5 exonuclease (T5exo) to the N-terminus of ErCas12a. *T5exo-ErCas12a* mRNA displayed efficient indel mutagenesis as well as mediated efficient knockin without heatshock. Taken together, our findings expanded and simplified the application of ErCas12a and further revealed the broad potential of Cas12a-mediated genome editing in zebrafish. 

## 2. Materials and Methods

### 2.1. Zebrafish Husbandry 

All zebrafish used in this study were maintained at 28.5 °C in the Peking University zebrafish facility with a 14 h/10 h light/dark cycle. The wild-type strain was Tübingen (TU).

### 2.2. Donor Plasmid Construction

The MMEJ tdTomato knockin donors were constructed based on a published strategy [14]. Briefly, *Eco*R I and *Eag* I were used to digest pMD18T-tdTomato to obtain the backbone, and then two pairs of primers were annealed and extended to obtain DNA fragments containing homology with the backbone, the 25 bp homologous sequence flanking the endogenous target site, and an *hEMX1* Cas9 site for donor linearization in vivo. Fragments were ligated with the backbone through Gibson assembly. All primers used for donor construction are listed in Table S1.

To construct the *tbx2b* Cas9 R295H donor, the *sox10* PoR-NeG donor [24] was digested with *Kpn* I and *Avr* II to obtain the backbone. The *tbx2b* intron 3 sequence downstream of the *tbx2b* Cas9 site together with full exon 4, intron 4, and exon 5 up to the sequence encoding R295 was amplified from the wild-type zebrafish genome. The homologous sequence of the backbone adjacent to the *Kpn* I site was introduced in the F primer, while the R295H point mutation coding sequence was introduced in the R primer, to create tbx2b I. The remaining CDS downstream of the R295 coding sequence was amplified from 48 hpf (hours post-fertilization) zebrafish embryonic cDNA, which was named *tbx2b* II. The R295H coding sequence was introduced in the F primer with an overlap of 30 bp to *tbx2b* I, while the homologous sequence of the backbone adjacent to the *Avr* II site was introduced in the R primer. The two fragments were ligated into the backbone using Gibson assembly to obtain the *tbx2b* Cas9 R295H donor.

To construct the *tbx2b* Cas12a R295H donor, the *tbx2b* Cas9 R295H donor was digested with *Age* I and *Avr* II to obtain the backbone. The sequence downstream from the *tbx2b* Cas12a site to the end of the CDS was amplified from the *tbx2b* Cas9 R295H donor, which was named *tbx2b* III. Homologous sequences of both backbone ends were introduced in the primers. The fragment was ligated into the backbone using a Gibson assembly to obtain the *tbx2b* Cas12a R295H donor.

Donor construction using the Gibson assembly followed the manufacture’s protocol (M5510AA; NEB, Ipswich, MA, USA). Restriction enzymes were obtained from NEB, and high-fidelity versions were adopted if available. 

### 2.3. Synthesis of gRNA, Pre-crRNA, and mRNAs Encoding Cas9, ErCas12a, and T5exo-ErCas12a

The gRNAs were designed with CasOT (http://casot.cbi.pku.edu.cn/ (accessed on 31 December 2021)) [28]. Forward oligonucleotides containing a T7 promoter, gRNA target site, and partial scaffold sequence were designed for gRNA template synthesis through PCR amplification using the pUC19-scaffold as the template [13], along with a common reverse primer. 

The pre-crRNA transcription templates were prepared by the double ssODN annealing and extension method as described previously [22]. Specifically, an F primer containing a T7 promoter and the entire scaffold coding sequence was used as a universal primer. R primers contained the reverse complementary protospacer sequence and a 21-nt sequence complementary to the scaffold coding sequence of the F primer. The two primers were annealed and extended by PCR to generate the template for in vitro transcription of pre-crRNA.

The gRNAs and pre-crRNAs were synthesized by in vitro transcription with the T7 RiboMAX™ Express Large Scale RNA Production System (Promega, Madison, WI, USA) and purified by ethanol precipitation, or LiCl precipitation when specified. The pre-crRNA and gRNA target sequences are listed in Tables S2 and S3, and the primer information for template synthesis is listed in Table S4.

*Xba* I-linearized pT3TS-nCas9n, carrying a zebrafish codon optimized Cas9, was used as the template for in vitro transcription of *Cas9* mRNA using the mMessage mMachine T3 kit (Ambion, Austin, TX, USA) [29]. 

A chemically synthesized zebrafish codon optimized ErCas12a coding sequence (Ruibiotech, Beijing, China) was cloned into pT3TS to generate pT3TS-ErCas12a. A zebrafish codon optimized T5 exonulcease coding sequence (T5exo) with an Xten linker was further cloned into pT3TS-ErCas12a to generate the pT3TS-T5exo-ErCas12a plasmid. *Xba* I-linearized plasmids were used for in vitro transcription using the mMessage mMachine T3 kit (Ambion, Austin, TX, USA).

All mRNAs were purified by LiCl precipitation according to the manufacturer’s protocol (Ambion, Austin, TX, USA).

### 2.4. Microinjection of Zebrafish Embryos

To evaluate the phenotype of injected embryos and the indel efficiency of each target site, 1 nL of a solution containing 600 ng/μL *Cas9, ErCas12a*, or *T5exo-ErCas12a* mRNA, and 100 ng/μL gRNA or pre-crRNA was injected into the animal pole of one-cell stage zebrafish embryos. For knockin experiments using only Cas9 or ErCas12a variants, 1 nL of a solution containing 300 ng/μL *Cas9, ErCas12a*, or *T5exo-ErCas12a* mRNA, 100 ng/μL of each gRNA or pre-crRNA, and 15 ng/μL of donor plasmid was injected into the animal pole of one-cell stage zebrafish embryos. Knockin experiments using ErCas12a variants for genome cleavage and Cas9 for donor linearization utilized a solution of 300 ng/μL of *Cas9* mRNA, 300 ng/μL of *ErCas12a* or *T5exo-ErCas12a* mRNA, and 100 ng/μL each of gRNA and pre-crRNA. 

For heatshock after ErCas12a injection, embryos were placed in a 34 °C incubator for 1 h or 4 h during different stages. After heatshock, embryos were sorted for fertilization, and fertilized embryos were moved to a 28.5 °C incubator as normal. The death rate of embryos after heatshock was calculated at 24 hpf.

### 2.5. Target Site Efficiency Evaluation

Genomic DNA of injected embryos was extracted by two rounds of 15-min lysis with 50 mM NaOH. Then, pooled genomes of five individual embryos were used as a template to amplify the corresponding genomic region of the target site, and the PCR products were sent for Sanger sequencing. The sequencing results of injected embryos and uninjected wildtype controls were uploaded to https://tide.nki.nl (accessed on 31 December 2021) [30] for TIDE analysis of the mutation efficiency and indel pattern. It is reported that TIDE can determine the spectrum and frequencies of indels with a limit of sensitivity of ∼1–2% across various target regions in a pool of cells. When comparing targeting efficiency under different experimental conditions, at least 3 sets of independent sequencing results of different genomes were used for TIDE analysis and the average value was considered as the target site efficiency under the experimental conditions. For detailed analysis, PCR products were purified and cloned into pMD19-T (Takara Bio, Kusatsu, Japan) for single-clone sequencing. Clone sequencing results were aligned to the genome sequence for the analysis of efficiency and indel patterns. Primers for target site genome amplification and Sanger sequencing of PCR products are listed in Table S5.

### 2.6. Junction PCR and Sanger Sequencing

48–72 hpf F_1_ embryos were screened for the correct fluorescence pattern. Genomic DNA was extracted by NaOH lysis as described above. Then, pooled genomes from 3–5 individuals were used as a template for PCR amplification of the 5′- and 3′-junction fragments of the KI alleles using donor- and genome-specific primers (Table S6). The PCR products were analyzed by Sanger sequencing.

### 2.7. Imaging and Processing

For imaging, zebrafish embryos were anesthetized with 0.02% tricaine (ethyl 3-aminobenzoate methanesulfonate salt; MilliporeSigma, Burlington, MA, USA), and positioned in 3% methylcellulose (MilliporeSigma, Burlington, MA, USA). Brightfield images were taken using a stereomicroscope (Stemi 305; Zeiss, Jena, Germany) equipped with AxioCam 208 color (Zeiss, Jena, Germany), and the images were processed by the ZEN 3.1 (blue edition) software. For fluorescence imaging, zebrafish embryos were pre-treated with 0.0045% PTU (1-Phenyl-2-thiourea; MilliporeSigma, Burlington, MA, USA) to inhibit pigmentation. Prior to imaging, embryos were anesthetized with 0.02% tricaine (ethyl 3-aminobenzoate methanesulfonate salt; MilliporeSigma, Burlington, MA, USA), and positioned in 3% methylcellulose (MilliporeSigma, Burlington, MA, USA). Fluorescence images were taken using a compound microscope (AXIO Imager Z1; Zeiss, Jena, Germany) equipped with AxioCam MRm (Zeiss, Jena, Germany) and processed by the AxioVision Rel.4.8 software. 

### 2.8. Statistics

Investigators were not blinded during phenotype assessment. Unpaired two-tailed Student’s *t*-test was used to analyze the difference of indel mutation efficiency of the same site or sites in the same region. Each group contained 3 to 5 independent replicates. The Chi-square test or Fisher’s exact test was used to calculate *p* values between different injections. In all figures, “*” indicates *p* < 0.05, “**” indicates *p* < 0.01, “***” indicates *p* < 0.001, and “n.s.” indicates that the difference is not significant. 

## 3. Results

### 3.1. Optimization of Heatshock Conditions for the Application of ErCas12a mRNA in Zebrafish

Although ErCas12a was previously reported to be functional when injected as mRNA into zebrafish embryos after 34 °C heatshock for 4 h [11], its efficacy was only tested in a few loci, and the heatshock condition was not optimized. We synthesized zebrafish codon-optimized ErCas12a with SV40 nuclear localization signals at both N- and C-termini and tested its mutagenic efficiency by co-injecting *ErCas12a* mRNA and pre-crRNAs targeting the zebrafish *alb* (*albino*) locus into one-cell stage zebrafish embryos and performing heatshock in various stages (Figure S1A). Some embryos showed abnormal morphology or death after injection and heatshock, particularly when heatshock immediately followed injection (Figure S1B), indicating certain toxicity induced by heatshock treatment. At 3 dpf (days post-fertilization), embryos showed different levels of reduced pigmentation, resembling the *alb* biallelic mutation, and the reduction was more severe in heatshocked embryos compared with normally incubated embryos (Figure S1C,D), demonstrating temperature-dependent activity of ErCas12a. Molecular analysis revealed that among the three pre-crRNAs, only site 2 showed obvious mutagenic efficiency. We then quantified the indel efficiency of *alb*-pre-crRNA2 by TIDE in each group. We noticed that while injected embryos without heatshock displayed ~40% indel efficiency, 4 h heatshock during any stage resulted in a significant increase in the rate of mutagenesis (Figure S1E). Given the fact that 4 h heatshock in early stages negatively impacted survival and normal development of the injected embryos, to reduce the heatshock damage to the embryos while still maintaining ErCas12a targeting efficacy, we performed 34 °C heatshock for one-hour intervals from 1 hpf to 9 hpf and examined the mutagenesis efficiency at 2 dpf. Surprisingly, one-hour heatshock within 3 hpf to 8 hpf could retain a similarly high indel efficiency as the 4 h treatment (Figure S1F), indicating that relatively embryo-friendly 1 h heatshock at 34 °C is sufficient to maximize the activity of ErCas12a.

### 3.2. ErCas12a Could Achieve Efficient MMEJ-Mediated Knockin in Zebrafish

**Figure 1:**
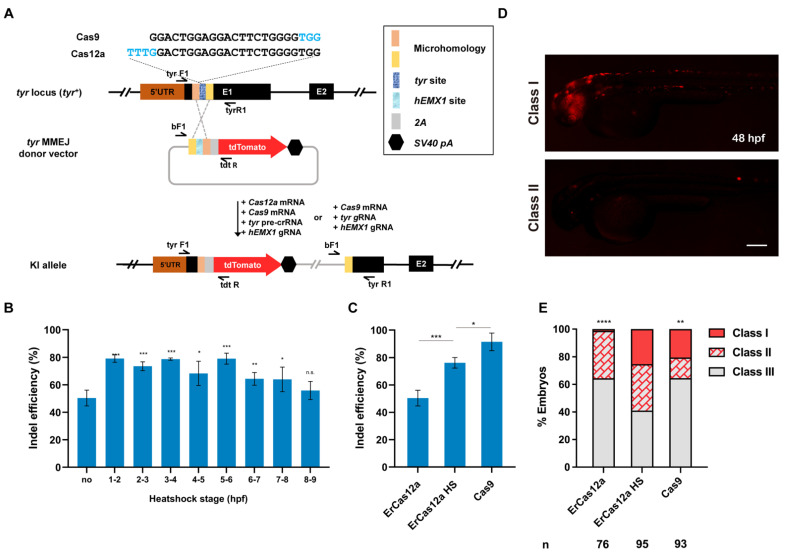
ErCas12a mediates efficient MMEJ-based knockin at the zebrafish *tyr* locus. (**A**) Schematic diagram of reporter gene knockin mediated by the MMEJ pathway through ErCas12a or Cas9 targeting at the *tyr* locus. The sequences of the overlapping target sites are displayed, with PAM sequences indicated in blue characters. (**B**) Indel efficiency of *tyr* ErCas12a site in embryos injected with *ErCas12a* mRNA and *tyr* pre-crRNA following 1 h heatshock at different developmental stages. (**C**) Comparison of indel efficiency of *tyr* Cas9 site with ErCas12a site with or without 1 h 34 °C heatshock at 3–4 hpf. HS: heatshock. Data in (**B**,**C**) represent mean ± s.d. of at least three independent replicates. Unpaired two-tailed Student’s *t*-test was used to calculate *p* values (* *p* < 0.05; ** *p* < 0.01; *** *p* < 0.001; “n.s.” indicates the difference is not significant). (**D**) Representative fluorescence expression of *tyr* knockin F_0_ embryos obtained by injection of either ErCas12a or Cas9 MMEJ systems. Embryos with red fluorescence were separated into broad (Class I) and sparse (Class II) groups according to the expression pattern. Embryos with no fluorescence were designated Class III. The embryos are in lateral view with anterior to the left. Scale bar: 200 μm. (**E**) Evaluation of MMEJ-based knockin efficiency by the proportion of broad (Class I) and sparse (Class II) fluorescence-positive embryos injected with ErCas12a or Cas9 MMEJ-based knockin system at the *tyr* locus. ErCas12a system injected embryos were separated into heatshocked (ErCas12a HS) and control (ErCas12a) groups. Number of embryos evaluated (n) is shown for each condition. Chi-square test was used to calculate *p* values between heatshocked (ErCas12a HS) group and others (** *p* < 0.01; **** *p* < 0.0001).

ErCas12a cleavage generally leaves sticky DNA ends, which is preferable to blunt ends for promoting homology-based DSB repair, though knockin applications have not been reported for ErCas12a. To evaluate whether ErCas12a is feasible for knockin and whether it could achieve efficiency comparable to Cas9, we identified an ErCas12a target site, which overlaps a highly efficient Cas9 target site in the *tyr* (*tyrosinase*) locus [29], and compared their performance in MMEJ-mediated knockin (Figure 1A). We first examined the indel efficiency of the *tyr* ErCas12a site under 1 h heatshock, and found that 1 h heatshock between 1 hpf and 6 hpf was sufficient to generate indel mutations up to 80%, slightly lower than the efficiency observed with Cas9 at the overlapping *tyr* Cas9 site (Figure 1B,C). We thus constructed a *tyr* MMEJ donor plasmid with an *hEMX1* Cas9 site for in vivo linearization, flanked by two 25-bp microhomology sequences, followed by a *tdTomato* reporter gene separated by a 2A peptide sequence (Figure 1A) [23,24,31]. Precise in-frame integration of the donor would lead to tdTomato expression under the control of the endogenous *tyr* promoter, as well as disruption of *tyr* gene function (Figure 1A). We co-injected *ErCas12a* mRNA, *tyr* pre-crRNA, *Cas9* mRNA, *hEMX1* gRNA, and the *tyr* MMEJ donor into one-cell stage zebrafish embryos and divided them into heatshock (34 °C at 3–4 hpf) and control (no heatshock) groups. A Cas9-based knockin system containing *Cas9* mRNA, *tyr* gRNA, *hEMX1* gRNA, and the *tyr* MMEJ donor was also injected for comparison of ErCas12a and Cas9 knockin efficiency. It has been reported that the distribution of fluorescence correlates well with knockin efficiency [11]. Thus, we qualitatively classified the injected embryos into three groups (Class I, II, and III) according to their fluorescence mosaicism. We clearly observed red fluorescent signals in each injected group at 48 hpf. In the ErCas12a heatshock group, nearly 60% (58/95) embryos showed scattered tdTomato expression, with around 25% of total embryos displaying a broad expression pattern (24/95) (Figure 1D,E). Since the broad expression pattern of the reporter gene in F_0_ embryos often correlates with high potential for germline transmission of knockin events [14,17,24], the knockin events generated by the ErCas12a with heatshock were likely to be heritable. In contrast, the ErCas12a control group without heatshock showed low knockin efficiency, with only 35% (27/76) of the embryos showing red fluorescence, and few embryos showed a broad pattern of tdTomato expression (Figure 1E), which is consistent with its lower indel mutagenesis efficiency (Figure 1B,C). In the group injected with the Cas9 knockin system, red fluorescent signals were observed in about 35% (33/93) of the embryos, and 20% (19/93) showed a broad pattern. Both ratios are lower than the ErCas12a heatshock group (Figure 1E), though the indel mutagenesis efficiency induced by Cas9 was higher than ErCas12a (Figure 1C). These results indicate that, like Cas9, ErCas12a is capable of promoting knockin through the MMEJ pathway.

**Figure 2:**
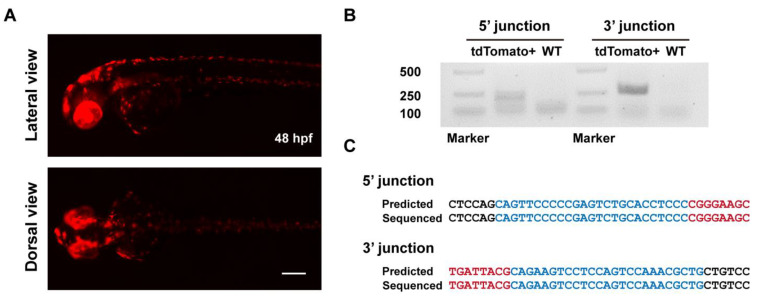
Evaluation of germline transmission of ErCas12a-mediated knockin with the MMEJ donor at the *tyr* locus. (**A**) Representative images of the red fluorescent reporter expression of F_1_ embryos obtained from outcross of the three *tyr* MMEJ knockin-positive F_0_ adults listed in Table 1. Red fluorescence can be observed in the eyes and pigment cells of the embryos. Scale bar: 200 μm. (**B**) Junction PCR results of the *tyr* knockin F_1_ embryos showing red fluorescence from F_0_ #3. Primers tyr F1 and tdt R were used to amplify the 5′ junction, and primers bF1 and tyr R1 were used to amplify the 3′ junction. (**C**) Sequencing alignment of the junction PCR products from (**B**) after TA cloning. Black characters indicate sequence of the *tyr* WT allele, blue characters represent the homologous sequence designed in the donor, and red characters represent the donor sequence.

To evaluate the heritability of ErCas12a-mediated knockin events, we raised the F_0_ embryos with a broad red fluorescence pattern from the ErCas12a heatshock group to maturity and carried out germline transmission analysis via an outcross with wildtype fish and observing tdTomato expression in the offspring. We successfully detected germline transmission in 42.9% (3/7) of the screened F_0_ fish (Figure 2A), with germ cell mosaicism in individual fish ranging from 15.4% to 34.0% (Table 1). To validate germline transmission at the molecular level, we extracted genomic DNA of F_1_ embryos with or without red fluorescence and performed junction PCR analysis using pairs of primers spanning the 5′ or 3′ junction of the integration site. The amplification results confirmed the successful integration of the MMEJ donor (Figure 2B). Since junction PCR cannot exclude the possibility of NHEJ-mediated integration of the donor, and MMEJ-mediated repair might be imprecise, to examine whether the integration of our *tyr* MMEJ donor was precise, we performed TA cloning and Sanger sequencing of the junction PCR products of the F_1_ genomes from each F_0_ fish. Sequencing results demonstrated that both ends of the donor were accurately integrated into the target site in the F_1_ embryos of each F_0_ (Figure 2C). These results showed that MMEJ-mediated knockin by *ErCas12a* mRNA is not only efficient, but also precise and highly heritable.

To evaluate whether ErCas12a-mediated efficient knockin via MMEJ was generally applicable, we selected the previously tested *alb* pre-crRNA2 and constructed the *alb* Cas12a MMEJ vector. Since an overlapping Cas9 target site with the *alb* pre-crRNA2 site was not identified, we chose a previously reported highly efficient Cas9 site whose cleavage point was located about 35 bp upstream of *alb* pre-crRNA2 for comparison (Figure S2A) [26]. We constructed an *alb* Cas9 MMEJ vector based on the local sequence information (Figure S2A). We then performed injection and treatment as before at the *tyr* locus. Efficient knockin, as shown by fluorescence expression, was observed with both ErCas12a and Cas9 injection, and the ErCas12a with the heatshock group showed more efficient knockin than the ErCas12a control (no heatshock) group, as expected (Figure S2B,C). Germline transmission was detected in one out of three screened F_0_ fish raised from ErCas12a-injected embryos showing a broad expression pattern of the reporter gene (Table 1). The 5′ junction of the inherited allele contained two point mutations whose sequence was identical to the PAM of the *hEMX1* donor linearization site (Figure S2D–F), which might be due to the error-prone recognition and repair mediated by DNA polymerase θ during MMEJ-mediated repair [32,33,34]. Moreover, no obvious ectopic expression of the fluorescent reporter was observed when embryos were co-injected with *Cas9* mRNA and *hEMX1* gRNA and different donors (Figure S2G). Together, these data demonstrated that ErCas12a with limited heatshock could induce MMEJ-mediated large fragment knockin more efficiently than Cas9 in some loci.

### 3.3. ErCas12a Could Efficiently Induce NHEJ-Based Knockin in Zebrafish

**Figure 3:**
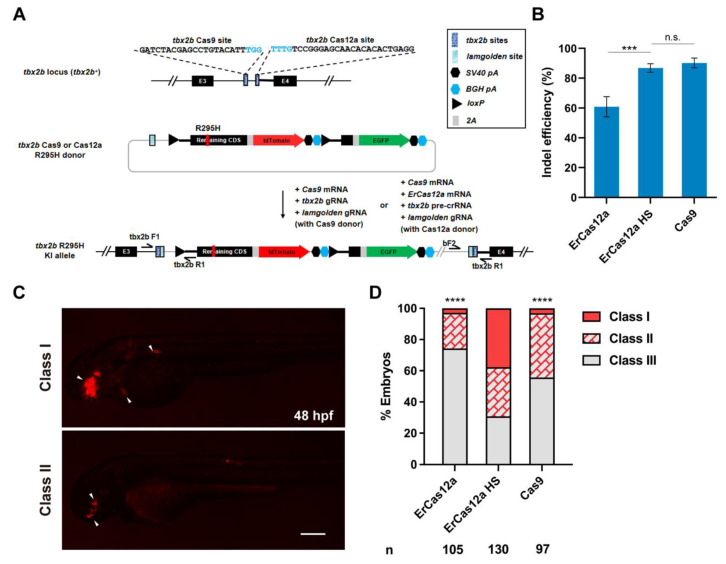
ErCas12a mediates efficient NHEJ-based knockin at the zebrafish *tbx2b* locus. (**A**) Schematic diagram of R295H mutation and reporter gene knockin mediated by the NHEJ pathway through ErCas12a or Cas9 targeting at the *tbx2b* locus. Target site sequences are displayed, with PAM sequences indicated in blue. (**B**) Comparison of indel efficiency of *tbx2b* Cas9 site with ErCas12a site with or without 1 h heatshock at 34 °C. HS: heatshock. Data represent mean ± s.d. of at least three independent replicates. Unpaired two-tailed Student’s *t*-test was used to calculate *p* values (*** *p* < 0.001; “n.s.” indicates the difference is not significant). (**C**) Representative fluorescence expression of *tbx2b* knockin F_0_ embryos obtained by injection of either ErCas12a or Cas9 NHEJ systems. Embryos with red fluorescence were separated into broad (Class I) and sparse (Class II) groups according to the expression pattern. Embryos with no fluorescence were designated Class III. The embryos are in lateral view with anterior to the left. Arrows indicate the regions showing red fluorescent signal. Scale bar: 200 μm. (**D**) Evaluation of NHEJ-based knockin efficiency by the proportion of broad (Class I) and sparse (Class II) fluorescence-positive embryos injected with ErCas12a or Cas9 systems at the *tbx2b* locus. The ErCas12a system injected embryos were separated into heatshocked (ErCas12a HS) and control (ErCas12a) groups. Number of embryos evaluated (n) is shown for each condition. Chi-square test was used to calculate *p* values between heatshocked (ErCas12a HS) group and others (**** *p* < 0.0001).

Next, we wondered whether ErCas12a could also achieve large fragment gene knockin via the NHEJ pathway as has been reported with Cas9. To evaluate the performance of ErCas12a in NHEJ-based knockin, we identified high activity Cas9 and ErCas12a target sites in the third intron of *tbx2b* locus (Figure 3A), with the ErCas12a site located ~260 bp downstream of the Cas9 site. After co-injection of *tbx2b* pre-crRNA and *ErCas12a* mRNA and 1 h heatshock at 34 °C, indel efficiency increased to 87% from 60% without heatshock, which is comparable to the 90% indel efficiency induced by Cas9 (Figure 3B). For comparison of NHEJ-based knockin efficiency, we constructed *tbx2b* Cas9 and Cas12a R295H donors based on our previously reported geno-tagging vector (Figure 3A) [24]. Each donor retained the intron as well as the splice acceptor sequence downstream of the *tbx2b* Cas9 or ErCas12a sites to facilitate splicing of the knockin allele. After injection, the donors would be linearized in vivo through the *lamgolden* Cas9 site and integrated into the *tbx2b* intron 3 through the NHEJ pathway. After transcription of the knockin allele, the downstream elements from the donor would be spliced into the third exon of *tbx2b* to generate a complete *tbx2b* coding sequence, but with the arginine encoding codon at position 295 mutated to a histidine codon, along with an in-frame tdTomato coding sequence, separated by a 2A peptide (Figure 3A). We first co-injected *Cas9* mRNA, *tbx2b* gRNA, *lamgolden* gRNA, and a *tbx2b* Cas9 R295H donor into one-cell stage zebrafish embryos for Cas9-mediated knockin and examined tdTomato expression at 2 dpf. In total, 44.3% (43/97) of the embryos showed a red fluorescent signal in the eyes, branchial arch, or heart, corresponding to the expected *tbx2b* expression pattern. However, only three embryos (3.1%) showed a relatively broad expression pattern (Figure 3C,D). We next co-injected *ErCas12a* mRNA, *Cas9* mRNA, *tbx2b* pre-crRNA, *lamgolden* gRNA, and the *tbx2b* Cas12a R295H donor into embryos for ErCas12a-mediated knockin, and divided them into heatshock and control groups. Surprisingly, 69.2% (90/130) of the injected embryos showed the correct red fluorescence expression in the heatshock group, with 49 embryos (37.6%) among them displaying a broad expression pattern, clearly indicating higher knockin efficiency than Cas9 (Figure 3D). 

**Figure 4:**
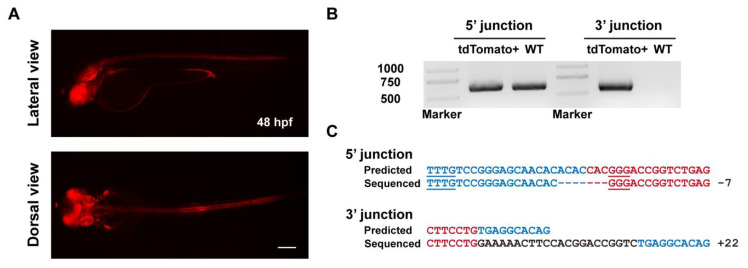
Evaluation of germline transmission of ErCas12a-mediated knockin with the NHEJ donor at the *tbx2b* locus. (**A**) Fluorescence reporter expression of F_1_ embryos obtained from outcross of a *tbx2b* NHEJ knockin F_0_ adult. Red fluorescence can be observed in the nervous system, eyes, pectoral fins, and endocardium of the F_1_ embryos. Scale bar: 200 μm. (**B**) Junction PCR results of the *tbx2b* knockin F_1_ embryos bearing red fluorescence. Primers tbx2b F1 and tbx2b R1 were used to amplify the 5′ junction, and primers bF2 and tbx2b R1 were used to amplify the 3′ junction. Note that due to the structure of the knockin allele, the 5′ junction amplicon of the of knockin allele is indistinguishable from that of the WT allele by electrophoresis. (**C**) Sequencing alignment of the junction PCR products after TA cloning. As the 5′ junction PCR product was a mixture of amplicons from the WT and knockin alleles, only sequencing results with indel mutations in the intronic junction site were considered to be amplified from the knockin allele. Blue characters indicate the *tbx2b* WT allele, red characters represent the donor sequences, and black characters represent random insertions resulting from NHEJ repair. Underlined characters indicate the PAMs of the *tbx2b* ErCas12a site and *lamgolden* Cas9 site for donor linearization.

Subsequently, we identified germline transmission of the *tbx2b* knockin alleles generated by ErCas12a with heatshock. Among 6 F_0_ fish, the progeny of one fish showed expected red fluorescent signals in the eyes, nervous system, and endocardium (Figure 4A, Table 1). The germ cell mosaicism (represented by the percentage of red fluorescence-positive F_1_ embryos) of this founder fish is 17.7% (6/34). Genotyping by junction PCR, TA cloning, and Sanger sequencing further identified correct and heritable integration of the donor into the *tbx2b* ErCas12a site (Figure 4B,C). Together, these results demonstrated that ErCas12a could efficiently mediate heritable NHEJ-based large fragment knockin under heatshock conditions.

We further sought to explore whether we could use ErCas12a rather than Cas9 for donor in vivo linearization to simplify the injection system. Since the *tbx2b* Cas9 R295H donor already contains the *tbx2b* ErCas12a target site as part of its downstream supplementary intronic sequence, we directly used this donor for our experiment. Curiously, after injection of *ErCas12a* mRNA, *tbx2b* pre-crRNA, and the *tbx2b* Cas9 R295H donor followed by heatshock, no tdTomato expression was observed in the embryos, and genomic integration was also not detected by junction PCR (data not shown). The addition of *Cas9* mRNA and *lamgolden* gRNA into the above injection system showed no improvement. Inspired by our previous findings on Cas9-mediated donor in vivo linearization [24], we adopted LiCl precipitation in place of ethanol precipitation to obtain LiCl-purified *tbx2b* pre-crRNA, and co-injected it with *ErCas12a* mRNA and the *tbx2b* Cas9 R295H donor (Figure S3A). After heatshock, 51.4% (57/111) of embryos showed mosaic red fluorescence, among which 19 embryos (17.1%) displayed a broad expression pattern (Figure S3B). Thus, we could achieve simultaneous genome cleavage and donor linearization with ErCas12a to induce NHEJ-mediated large DNA fragment knockin with a simple injection system.

### 3.4. T5exo-ErCas12a Efficiently Induces Indel Mutation and Promotes Knockin while Avoiding Heatshock Treatment in Zebrafish

In the previous sections, we showed that ErCas12a could mediate large fragment knockin by the MMEJ and NHEJ pathways. However, a 1 h heatshock treatment of the injected embryos was necessary to achieve high efficiency. It would be ideal to further modify ErCas12a as a heatshock-free tool for convenient and embryo-friendly applications. Previous reports suggested that co-transfection of DNA exonuclease with Cas9 in human induced pluripotent stem cells increased the mutagenesis rate [35], while the fusion of the DNA exonuclease to the N-terminus or C-terminus of FnCas12a improved its gene editing efficiency to a similar or even higher level compared to those of LbCas12a and AsCas12a [36]. Thus, we first employed the commonly used human 3′ exonuclease TREX2 and fused it to either the N- or C-terminus of ErCas12a and tested its efficiency using *tyr* pre-crRNA and *alb* pre-crRNA. Unexpectedly, TREX2 fused to either end of ErCas12a displayed lower mutagenesis compared to ErCas12a without heatshock (Figure S4A–F), and C-terminal fusion even dramatically decreased the indel efficiency with heatshock (Figure S4A–C), which suggested that, unlike Cas9, ErCas12a was not suitable for co-application with TREX2 to improve its mutagenesis.

**Figure 5:**
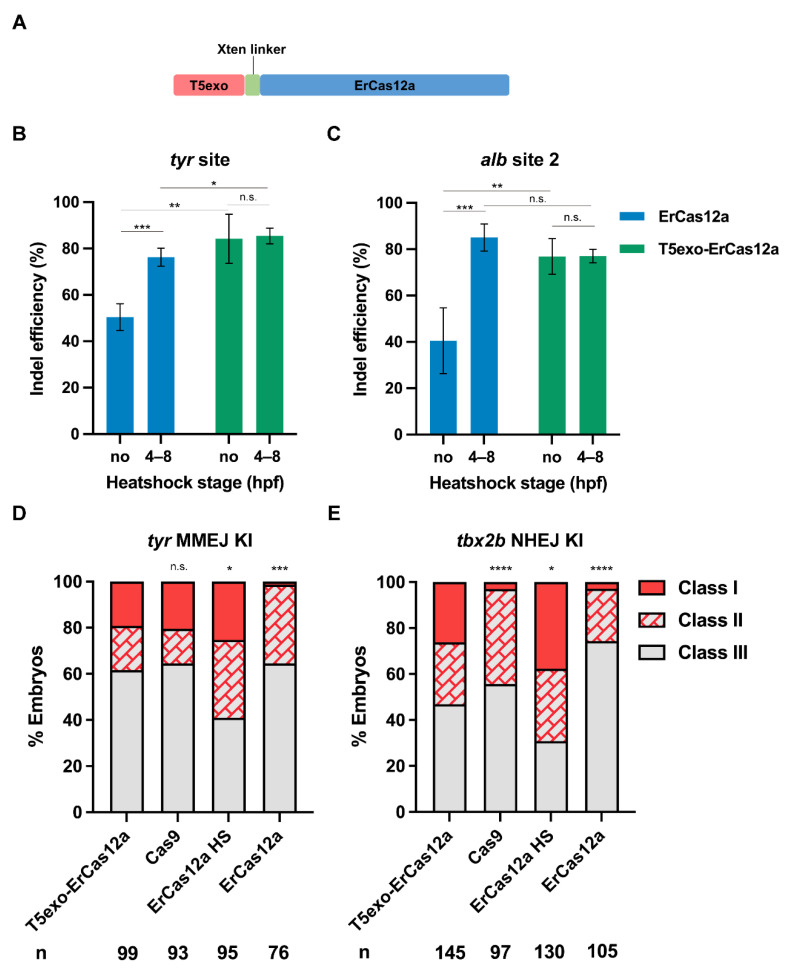
T5exo-ErCas12a efficiently mediates heatshock-free indel mutation and large fragment knockin. (**A**) Schematic diagram of ErCas12a fused with T5 exonuclease at the N-terminus. The two proteins are separated by an Xten linker. (**B**) Indel efficiency in embryos injected with *T5exo-ErCas12a* mRNA and *tyr* pre-crRNA under heatshock or non-heatshock conditions. The results were compared with *ErCas12a* mRNA injection under the same conditions. (**C**) Indel efficiency in embryos injected with *T5exo-ErCas12a* mRNA and *alb* pre-crRNA2 under heatshock or non-heatshock conditions. The results were compared with *ErCas12a* mRNA injection under the same conditions. Data in (**B**,**C**) represent mean ± s.d. of at least three independent replicates. Unpaired two-tailed Student’s *t*-test was used to calculate *p* values (* *p* < 0.05; ** *p* < 0.01; *** *p* < 0.001; “n.s.” indicates the difference is not significant). (**D**) Evaluation of MMEJ-based knockin efficiency by the proportion of broad (Class I) and sparse (Class II) fluorescence-positive embryos injected with T5exo-ErCas12a, Cas9, or ErCas12a (with and without heatshock) systems at the *tyr* locus. Representative images of different expression patterns are shown in Figure 1D. HS: Heatshock. (**E**) Evaluation of NHEJ-based knockin efficiency by the proportion of broad (Class I) and sparse (Class II) fluorescence-positive embryos injected with T5exo-ErCas12a, Cas9, or ErCas12a (with and without heatshock) systems at the *tbx2b* locus. Representative images of different expression patterns are shown in Figure 3C. HS: Heatshock. Number of embryos evaluated (n) is shown for each condition. Chi-square test was used to calculate *p* values between T5exo-ErCas12a group and others in (**D**,**E**) (* *p* < 0.05; **** *p* < 0.0001; “n.s.” indicates the difference is not significant).

Considering that ErCas12a generates 5′ protruding sticky ends at the site of the DSB, we wondered whether a 5′ exonuclease could improve editing efficiency. Encouraged by the report that T5 exonuclease fusion at the N-terminus of LbCas12a increased mutagenic efficiency in plants [37], we fused zebrafish codon optimized T5 exonuclease to the N-terminus of ErCas12a using an Xten linker (Figure 5A), and tested *tyr* and *alb* for indel efficiency. At both target sites and regardless of whether heatshock was performed, most injected embryos showed a distinct lack of pigmentation at 2 dpf. The mutation efficiency assay at the molecular level indicated that indel efficiency of the heatshock and non-heatshock groups after *T5exo-ErCas12a* mRNA injection were both nearly equal to that of *ErCas12a* mRNA injection with heatshock at both target sites (Figure 5B,C). We also performed injection of *T5exo-ErCas12a* mRNA and *tyr* pre-crRNA and evaluated embryo mortality at 24 hpf. No notable increase in mortality for the injected embryos compared with the uninjected control was observed (Figure S5). These results indicated that ErCas12a fused with T5 exonuclease at the N-terminus could achieve highly efficient genome editing without the need for heatshock, which meant that T5 exonuclease fusion improved the mutagenic activity of ErCas12a in normal (28.5 °C) zebrafish growing conditions.

Since T5exo-ErCas12a displayed efficient indel mutagenesis without heatshock, we further examined its performance for knockin under non-heatshock conditions. First, we performed MMEJ-based knockin experiments at the *tyr* locus using *T5exo-ErCas12a* mRNA to replace *ErCas12a* mRNA in the injection system. After injection without heatshock, we found that 38.4% (38/99) of the embryos showed tdTomato expression in pigment cells, and 19.2% (19/99) of the embryos displayed a broad expression pattern (Figure 5D). Genomic qPCR results showed that our classification of founder embryos by fluorescence patterns strongly correlates with the efficiency of donor integration (Figure S6). Next, in order to test whether T5exo-ErCas12a is suitable for knockin through the NHEJ pathway, we performed the knockin experiment at the *tbx2b* locus in a similar manner. The *tbx2b* Cas12a R295H donor was co-injected with *Cas9* mRNA, *T5exo-ErCas12a* mRNA, *tbx2b* pre-crRNA, and *lamgolden* gRNA into embryos followed by normal incubation. At 2 dpf, 53.1% (77/145) of the embryos showed the correct red fluorescence pattern, among which 38 (26.2%) showed a broad pattern (Figure 5E), indicating that the NHEJ-mediated knockin efficiency of T5exo-ErCas12a without heatshock was also similar to that of ErCas12a under heatshock. Therefore, T5 exonuclease fused ErCas12a could efficiently achieve large fragment gene knockin through either MMEJ or NHEJ pathways in zebrafish without the need for the damaging 34 °C heatshock. 

Given the high indel efficiency of the *T5exo-ErCas12a* mRNA without heatshock at both *tyr* and *alb* sites, it is difficult to evaluate the effect of heatshock on T5exo-ErCas12a. In order to explore whether the genome editing efficiency of our T5exo-ErCas12a could be further increased by heatshock, we evaluated an ErCas12a site in the fourth intron of *egfl7* (Figure S7A). Co-injection of *egfl7* pre-crRNA with *ErCas12a* mRNA and heatshock induced an indel efficiency of 54%, while the efficiency without heatshock was much lower (Figure S7B). However, when *egfl7* pre-crRNA was co-injected with *T5exo-ErCas12a* mRNA, the indel efficiency of the embryos incubated with or without heatshock only showed a mild difference (Figure S7B), indicating that the impact of heatshock on the genome editing activity of T5exo-ErCas12a at relatively low-efficiency sites is limited.

## 4. Discussion

In this study, we explored genome editing with ErCas12a in zebrafish for both mutagenic knockout and large fragment knockin. While previous reports suggested that ErCas12a could function in the mRNA form in zebrafish embryos with a 4 h heatshock [11], we found that only one-hour heatshock was sufficient for high efficiency ErCas12a editing, reaching about 80% indel efficiency with *tyr* or *alb* pre-crRNA. Encouraged by this, we performed large fragment knockin via MMEJ and NHEJ pathways and found that heatshock-dependent ErCas12a-mediated knockin was highly efficient, heritable, and comparable with Cas9-mediated knockin experiments. Furthermore, the fusion of T5 exonuclease to the N-terminus of ErCas12a successfully induced efficient heatshock-free gene knockout and knockin, which is the first CRISPR/Cas12a tool reported to be suitable for application under normal zebrafish embryo culture conditions, to our knowledge. In summary, our results suggested that ErCas12a and T5exo-ErCas12a could mediate efficient genome editing in mRNA form in zebrafish under convenient, embryo-friendly experimental conditions, and could complement or substitute Cas9 and expand the toolbox for genome editing in zebrafish as well as other species, especially for those that could not endure heatshock treatment. 

While Cas9 in zebrafish is already well-established in numerous applications [12,13,14,15,16,17,18,19,23,24,25,26,27,29,38], the use of Cas12a in zebrafish was not reported until 2017, and has not been widely adopted [11,21,33]. One major issue for the limited use of Cas12a is that AsCas12a and LbCas12a are only reported to be functional in the RNP form in zebrafish. Thus, ErCas12a likely has better application potential, as it can function in the mRNA form [11], and through modification with the T5 exonuclease can function without heatshock, and we have indeed demonstrated its efficacy in MMEJ- and NHEJ-based large fragment gene knockin. 

As LbCas12a has more potential target sites than Cas9 within the zebrafish genome [33], and ErCas12a possesses less-strict PAM requirements compared to LbCas12a [11,38], ErCas12a may therefore expand the targetable range across the whole zebrafish genome, which is important and sometimes essential for diverse applications of genome manipulation in zebrafish. Moreover, the GC ratio of zebrafish introns is extremely low [39], which limits the applicability of Cas9 for intronic targeting due to its GC-rich PAM and requirement of relatively high GC content of the protospacer sequence [40,41]. In contrast, ErCas12a with a TA-rich PAM may be more suitable for intron-targeting knockin methods [16,17,23,24]. In our experiments, both exonic and intronic ErCas12a target sites could achieve indel efficiency as high as 80% and were suitable for large fragment knockin, indicating the impressive potential of ErCas12a for advanced and complex genome manipulation experiments.

Theoretically, Cas12a may mediate homology-based knockin more efficiently than Cas9 [10,11], as the DSBs produced by Cas12a have sticky ends compared to the blunt-end DSBs produced by Cas9. Indeed, our MMEJ-mediated knockin results showed that ErCas12a with heatshock could mediate donor integration with higher efficiency than Cas9 at two sites tested, which is consistent with this notion [11]. Interestingly, NHEJ-based knockin experiments at the *tbx2b* locus also indicated that ErCas12a had higher knockin efficiency than a nearby Cas9 site. As the donor had no homologous sequences flanking the donor linearization site, we considered integration to occur via NHEJ, though we could not rule out the possibility that MMEJ-associated factors may participate in the knockin process. However, Sanger sequencing results of the junction PCR products did not show obvious microhomology-based repair, suggesting that there might be currently unknown mechanisms for the highly efficient NHEJ-mediated integration at ErCas12a-targeted sites. Moreover, whether ErCas12a generally performs better than Cas9 or not requires more validation to exclude potential context- or sequence-specific factors. Nevertheless, our results clearly indicate that ErCas12a could efficiently mediate large fragment gene knockin through both MMEJ and NHEJ pathways and could be used as an alternative or a supplement to Cas9.

Optimization of the heatshock requirements for Cas12a-mediated gene editing or eliminating the necessity of heatshock through the use of T5exo-ErCas12a could improve research throughput by simplifying procedures and reducing embryo death. Furthermore, it is possible that T5exo-ErCas12a could benefit applications where CRISPR gene editing is used in conjunction with heatshock promoter constructs, which are widely used in zebrafish research [42,43,44,45]. This additional highly active CRISPR system could also advantage applications where orthogonal Cas proteins are used simultaneously for different effects.

While T5exo-ErCas12a did not require heatshock for efficient genome editing, heatshock also did not further improve its indel mutagenesis activity. DNA exonuclease is expected to function after endonuclease cleavage of genomic DNA, causing deletions of the free ends and preventing precise repair, thus increasing mutagenesis. However, we found no notable increase in large deletions at the *tyr* or *alb* sites in the T5exo-ErCas12a injected embryos (Figure S8). Interestingly, the indel efficiency of T5exo-ErCas12a without heatshock and ErCas12a with heatshock were similar at each target site. Thus, the T5 exonuclease fused to ErCas12a may not solely function as an exonuclease but may also participate in other processes during genome editing, such as loosening the chromatin structure or unwinding the DNA double helix. Further explorations are required to reveal the molecular mechanism of T5exo-ErCas12a in DNA cleavage and repair.

## 5. Conclusions

Our results showed that ErCas12a and T5exo-ErCas12a can induce efficient gene knockout and large fragment gene knockin via MMEJ and NHEJ pathways when injected in RNA form in zebrafish. While high-activity genome editing using ErCas12a requires heatshock for 1 h at 34 °C, T5exo-ErCas12a omits the necessity of heatshock treatment. In addition, both ErCas12a with heatshock and T5exo-ErCas12a without heatshock display efficient and heritable knockin by MMEJ and NHEJ similar to Cas9 in the same target region. Together, we demonstrated that both ErCas12a and T5exo-ErCas12a could mediate efficient genome editing and expanded the engineered endonuclease toolbox in zebrafish. 

## Figures and Tables

**Table 1:** Germline transmission of ErCas12a MMEJ knockin positive F_0_ adults.

Gene	F_0_	Sex	Number of tdTomato-Positive F_1_ Embryos	Number of Total F_1_ Progeny	F_0_ Germ Cell Mosaicism (Ratio of Positive F_1_)
*tyr*	#3	Male	35	103	34.0%
	#5	Male	27	82	33.0%
	#7	Male	4	26	15.4%
*tbx2b*	#1	Male	6	34	17.7%

## Data Availability

All data generated or analyzed during this study are included in the manuscript and supplementary materials.

## Supplementary Materials

The following are available online at https://www.mdpi.com/article/10.3390/biology11030411/s1. Table S1. Primers for donor plasmid construction in this study; Table S2. The ErCas12a pre-crRNA target sequences used in this study; Table S3. The SpCas9 gRNA target sequences used in this study; Table S4. Primers for pre-crRNA and gRNA in vitro transcription template synthesis; Table S5. Primers for target site amplification and Sanger sequencing in this study; Table S6. Primers for knockin junction PCR in this study; Table S7. Primers for genomic qPCR used in this study. Figure S1. Evaluation of ErCas12a mutagenesis capacity at the zebrafish alb locus; Figure S2. ErCas12a mediates efficient MMEJ-based knockin at the zebrafish alb locus; Figure S3. ErCas12a could simultaneously achieve genome cleavage and donor linearization and mediate NHEJ-based knockin at the tbx2b locus; Figure S4. Mutagenesis capacity of N- or C- terminal fusion of TREX2 to ErCas12a; Figure S5. Evaluation of the embryo mortality after T5exo-ErCas12a system injection; Figure S6. Evaluation of KI efficiency among different fluorescent groups mediated by T5exo-ErCas12a; Figure S7. The effect of heatshock on T5exo-ErCas12a at the egfl7 locus; Figure S8. Evaluation of the indel mutation forms induced by T5exo-ErCas12a.
