# Supplementary material for: ErCas12a and T5exo-ErCas12a Mediate Simple and Efficient Genome Editing in Zebrafish
Source: Biology (Basel). 2022 Mar 8;11(3):411. doi: 10.3390/biology11030411 (PMC8945719; doi:10.3390/biology11030411)
Supplement: Supplementary file 1 [file biology-11-00411-s001.zip › biology-1383464-supplementary.pdf]

Table S1. Primers for donor plasmid construction used in this study

Donor fragment	Sequence
tbx2b I	F: GAGCGGCGCTTGTAATGGAAAGGTACCATTTGGTGGGATTTACTGTTTG<br>R: CTCATACATGTGTAATGATGGAAGGGTCAGCT
tbx2b II	F: CCATCATTACACATGTATGAGGATCAATGTAA<br>R: GTTAGAAGACTTCCTCTGCCCTCCCTAGGCTTGGGTGAATCCCTAGGTGAC
tbx2b III	F: CGGGTCAACGACTTCCTGCACGGGACCGGTCACAGCAATCAAAGCGTGTCAG<br>R: GTTAGAAGACTTCCTCTGCCCTCCCTAGGCTTGGGTGAATCCCTAGGTGAC
tyr MMEJ	F1: AGAAGTCCTCCAGTCCAAACGCTGGTCACCTCCAATGACTAGGGTGG<br>F2: GGAAACAGCTATGACCATGATTACGCAGAAGTCCTCCAGTCCAAACGCTG<br>R1: GGGGAGGTGCAGACTCGGGGGAACTGCCACCCTAGTCATTGGAGGTGAC<br>R2: GCTGAAGTTAGTAGCTCCGCTTCCCGGGGAGGTGCAGACTCGGGGGAACTG
alb Cas12a<br>*hEMX1* MMEJ	F1: CGCTGTTGAGGCTGCGTTTGTCACGTCACCTCCAATGACTAGGGTGG<br>F2: AGGAAACAGCTATGACCATGATTACCCTAGGACGCTGTTGAGGCTGCGTTTGTCAC<br>R1: GTAGCAGAATTCCCTTCCAAACATGGCCCACCCTAGTCATTGGAGGTGAC<br>R2: GGCTGAAGTTAGTAGCTCCGCTTCCCGGCCGGTAGCAGAATTCCCTTCCAAACATGGC

alb Cas9<br>*hEMX1* MMEJ	F1:<br>CACGGGTCGGCCATGTTTGGAAGGGGTCACCTCCAATGACTAGGGTGG<br>F2:<br>AGGAAACAGCTATGACCATGATTACCCTAGGCACGGGTCGGCCATGTTTGGAAGGG<br>R1:<br>ATAATCAACCTTCCCCTAGAGCGCCCACCCTAGTCATTGGAGGTGAC<br>R2:<br>GGCTGAAGTTAGTAGCTCCGCTTCCCGGCCGCATAATCAACCTTCCCCTAGAGCGC

Table S2. The ErCas12a pre-crRNA target sequences used in this study

Target gene	Location	Target sequence
alb (site 2)	exon 1	TTTGGAAGGGAATTCTGCTACGCTGTT (forward strand)
tyr	exon 1	CCACCCCAGAAGTCCTCCAGTCCAAA (reverse strand)
tbx2b	intron 3	TTTGTCCGGGAGCAACACACACTGAGG (forward strand)
egfl7	intron 4	TTTGTATGGTGGGACGTAAAACTTTCA (forward strand)

Note: the underlined sequence indicates PAM of the target site.

Table S3. The SpCas9 gRNA target sequences used in this study

Target gene	Location	Target sequence
alb	exon 1	GGGGAAGGTTGATTATGCACGGG (forward strand)
tyr	exon 1	CCACCCCAGAAGTCCTCCAGTCC (reverse strand)
tbx2b	intron 3	GATCTACGAGCCTGTACATTTGG (forward strand)
hEMX1	donor	GTCACCTCCAATGACTAGGGTGG (forward strand)
lamgolden	donor	GGGTCAACGACTTCCTGCACTGG (forward strand)

Note: the underlined sequence indicates PAM of the target site.

Table S4. Primers for pre-crRNA and gRNA *in vitro* transcription template synthesis

Primer	Application	Sequence information
T7-sfd Fwd	Cas9	TAATACGACTCACTATAGNNNNNNNNNNNNNNNNNNNGTTTTAGAGCTAGAAATAGC
Tracr Rev	Cas9	AAAAAAAGCACCGACTCGGTGCCAC
Precr F	ErCas12a	TAATACGACTCACTATAGGGTCAAAAGACCTTTTTAATTTCTACTCTTGTAGAT
Precr R	ErCas12a	NNNNNNNNNNNNNNNNNNNNNNNNATCTACAAGAGTAGAAATTAA

Note: the underlined sequence indicates T7 promoter, the Ns indicate the protospacer sequence of the corresponding target site (protospacer reverse complementary sequence for Precr R).

Table S5. Primers for target site amplification and Sanger sequencing used in this study

Target gene	Location	Primer sequence (Sanger sequencing primer is in bold)
tbx2b	intron 3	**tbx2b F1: ACTGCTTATCAAAACGACAAG**<br>tbx2b R1: CCATGAAAATAGAAAGCGATG
tyr	exon 1	tyr F1: GCGTCTCACTCTCCTCGACTCTTC<br>**tyr R1: GTAGTTTCCGGCGCACTGGCAG**
alb	exon 1	**alb F1: ATGACTCTTCTTACTGAGGACC**<br>alb R1: GTAGTTTCCGGCGCACTGGCAG
egfl7	intron 4	egfl7 F1: GTGGATCTAATTGAAGTAAC<br>**egfl7 R1: ACCGCTATATAGAGTGAGAC**

Table S6. Primers for knockin junction PCR used in this study

Primer name	Location	Sequence
tdt R	tdTomato coding sequence	CTTTGATGACCTCCTCGCCC
bF1 (M13-48)	donor plasmid backbone	GAGCGGATAACAATTTCACACAGG
bF2	donor plasmid backbone	CCTGCGTTATCCCCTGATTC

Table S7. Primers for genomic qPCR used in this study

Primer name	Location	Sequence
tyr F1	*tyr* exon 1	GCGTCTCACTCTCCTCGACTCTTC
tdt R	tdTomato coding sequence	CTTTGATGACCTCCTCGCCC
tyr geno qF	*tyr* exon 1	CTGCCAGTGCGCCGGAAACTACAT
tyr geno qR	*tyr* exon 1	GCGCGTACGTTCCTGTTACG

Supplementary figure legends

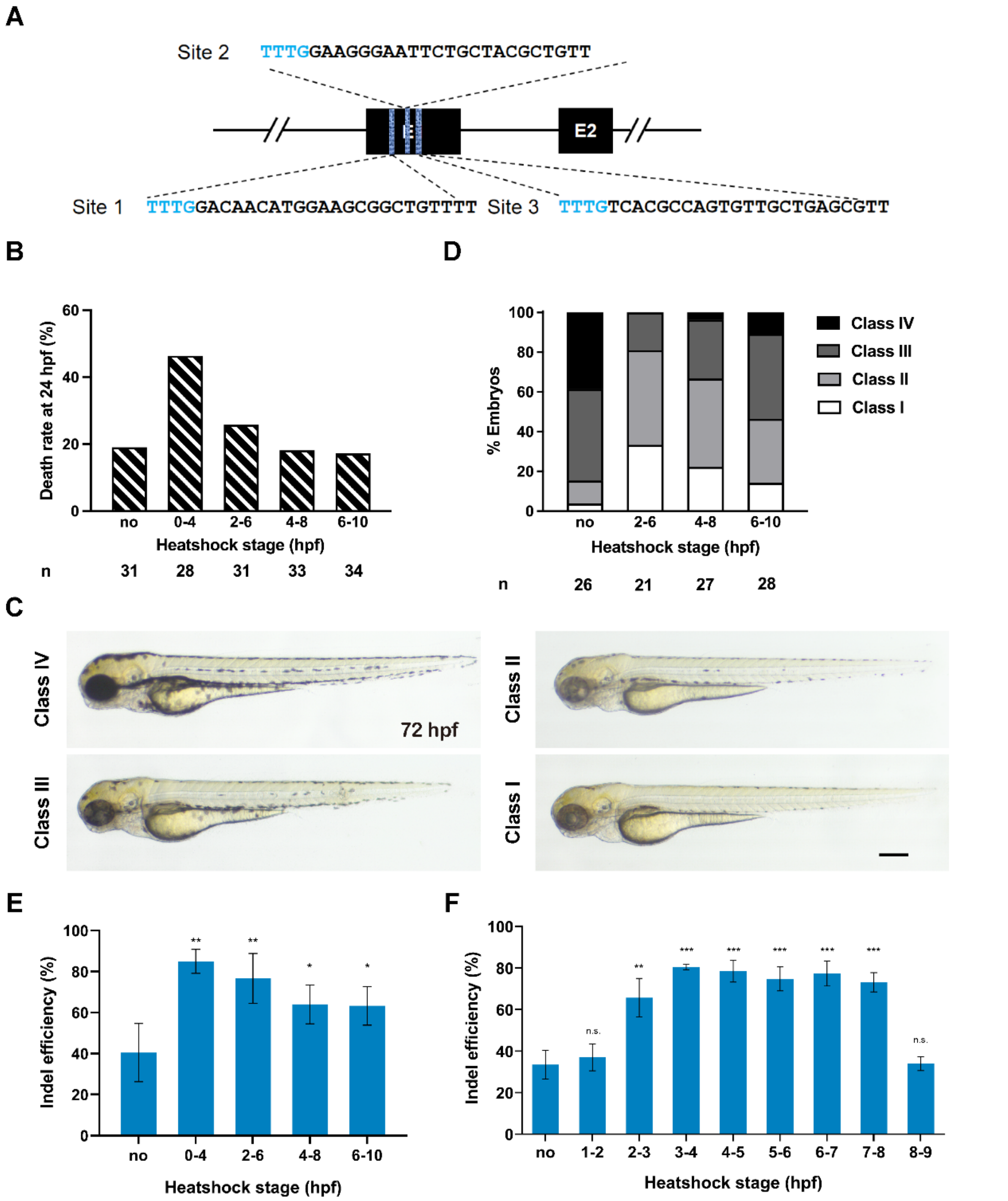

Figure S1. Evaluation of ErCas12a mutagenesis capacity at the zebrafish *alb* locus. (A) Schematic diagram of the location and sequence of the *alb* ErCas12a target sites. The PAM

sequence is indicated in blue characters. (**B**) Death rate of embryos injected with *ErCas12a* mRNA and *alb* pre-crRNAs after 4 h heatshock at 34°C in different developmental stages. Injected embryos without heatshock treatment were included as control. Number of embryos evaluated (n) is shown for each condition. (**C**) Classification of phenotype after injection of *ErCas12a* mRNA and *alb* pre-crRNAs. Representative images of different levels of mosaicism are shown. Scale bar: 200 μm. (**D**) Pigmentation phenotype evaluation of the injected embryos in each group treated with different heatshock conditions. Number of embryos evaluated (n) is shown for each condition. (**E**) Indel efficiency of *alb* site 2 in embryos injected with *ErCas12a* mRNA and *alb* pre-crRNA targeting site 2 after 4 h heatshock in different developmental stages. Injected embryos without heatshock treatment were provided as control. (**F**) Indel efficiency of *alb* site 2 in embryos injected with *ErCas12a* mRNA and *alb* pre-crRNA2 after 1 h heatshock in different developmental stages. Data represents mean +/- s.d. of at least 3 independent replicates. Unpaired two-tailed Student's *t*-test was used to calculate *p* values (* $p < 0.05$; ** $p < 0.01$; *** $p < 0.001$; "n.s." indicates the difference is not significant).

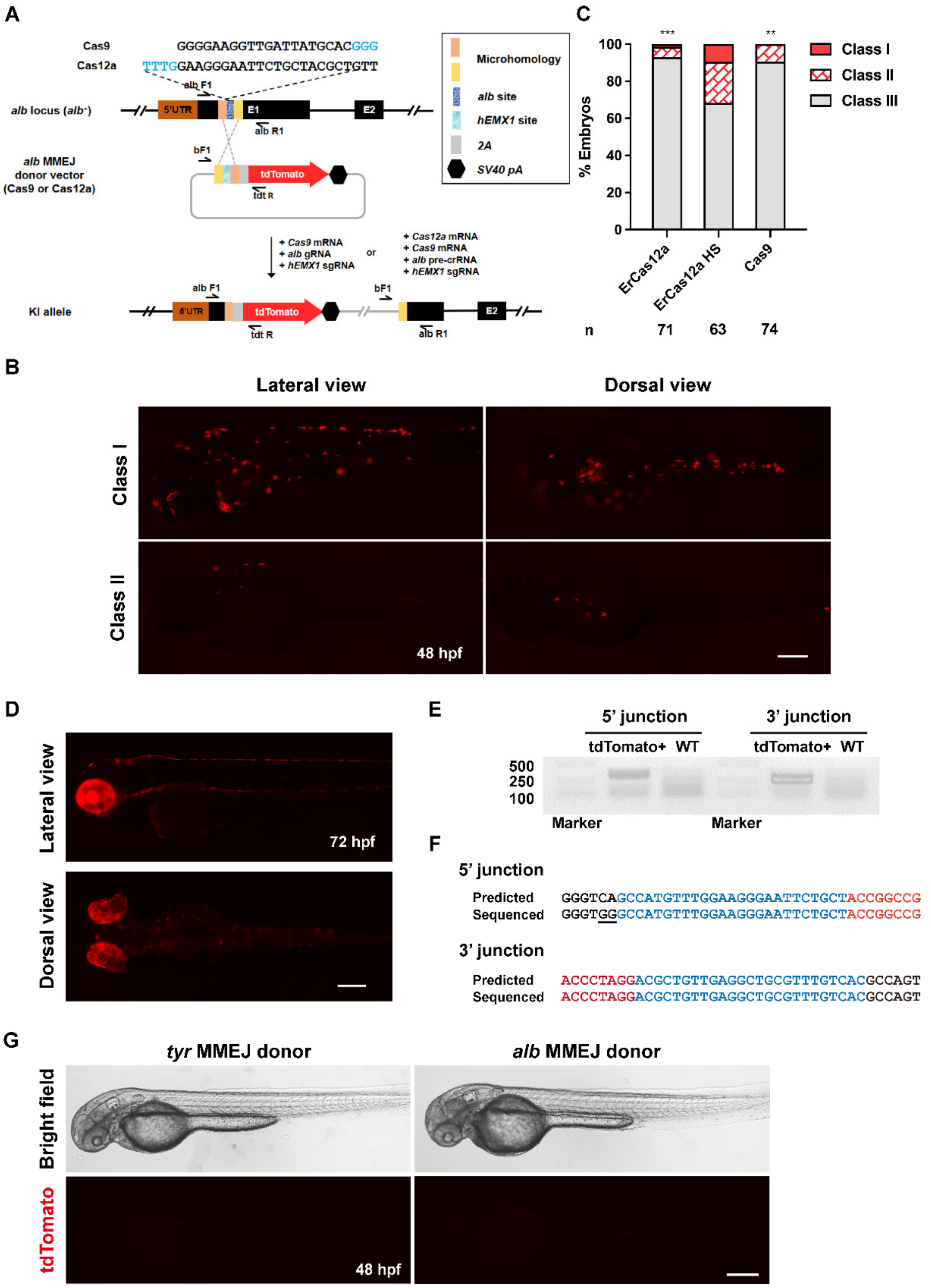

Figure S2. ErCas12a mediates efficient MMEJ-based knockin at the zebrafish *alb* locus. (**A**) Schematic diagram of reporter gene knockin mediated by the MMEJ pathway at the ErCas12a site 2 or Cas9 site in the *alb* locus. Target site sequences are displayed, and the PAM sequences are indicated in blue characters. Note that the *alb* Cas9 site and ErCas12a site 2 do not overlap, thus they were injected with different MMEJ donors. (**B**) Representative fluorescence expression of *alb* knockin F_0 embryos obtained by injection of either ErCas12a or Cas9 MMEJ system. Embryos with red fluorescence were categorized according to the

expression pattern. Scale bar: 200 µm. (**C**) Evaluation of MMEJ-based knockin efficiency by the proportion of class I and class II fluorescence-positive embryos injected with ErCas12a or Cas9 system at *alb* locus. ErCas12a system injected embryos were further separated into heatshocked (ErCas12a HS) and control (ErCas12a) groups. Number of embryos evaluated (n) is shown for each condition. Freeman-Halton extended Fisher's exact test was used to calculate *p* values between the ErCas12a HS group and others (** $p < 0.01$; *** $p < 0.001$). (**D**) Fluorescence expression of F_1 embryos obtained from outcross of an *alb* MMEJ knockin F_0 adult. Red fluorescence can be observed in the eyes and pigment cells. Scale bar: 200 µm. (**E**) Junction PCR results of the genome of *alb* knockin F_1 embryos bearing red fluorescence. Primers alb F1 and tdt R were used to amplify the 5' junction, and primers bF1 and alb R1 were used to amplify the 3' junction. (**F**) Sequencing result alignment of the junction PCR products after TA cloning. The black characters indicate the sequence of *alb* WT allele, the blue characters represent the homologous sequence designed in the donor, the red characters represent the donor-only sequence, and the underlined characters indicate the two consecutive point mutations different from the prediction. (**G**) Representative images of F_0 embryos obtained by injection of *Cas9* mRNA, *hEMX1* gRNA, and different donors (*tyr* MMEJ donor, *alb* MMEJ donor). No obvious ectopic expression of fluorescent reporter was observed in any embryo. Scale bar: 200 µm.

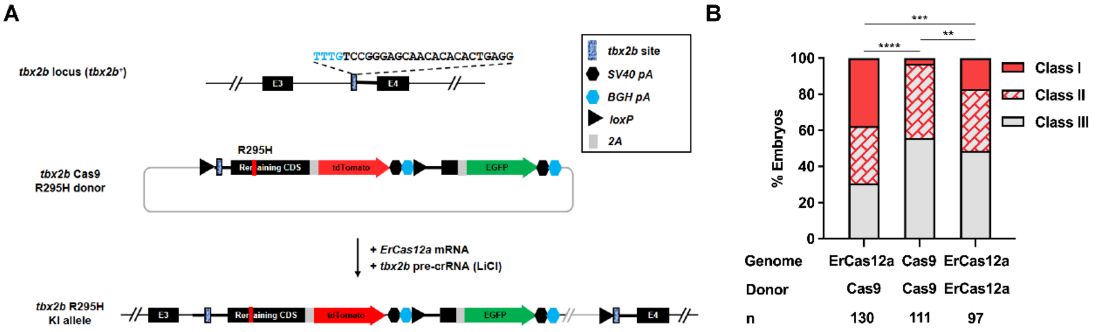

Figure S3. ErCas12a could simultaneously achieve genome cleavage and donor linearization and mediate NHEJ-based knockin at the *tbx2b* locus. (**A**) Schematic diagram of NHEJ-mediated knockin through *ErCas12a* mRNA and *tbx2b* pre-crRNA targeting both genome and donor for linearization. (**B**) Evaluation of NHEJ-based knockin efficiency by the proportion of class I and class II fluorescence-positive embryos injected with different systems using ErCas12a or Cas9 for either genome or donor cleavage. Note that all *ErCas12a* mRNA injected embryos displayed in this figure were treated with heatshock. Representative images of different expression patterns are shown in Figure 3C. Number of embryos evaluated (n) is shown for each condition. Chi-square test was used to calculate *p* values (** $p < 0.01$; *** $p < 0.001$; **** $p < 0.0001$).

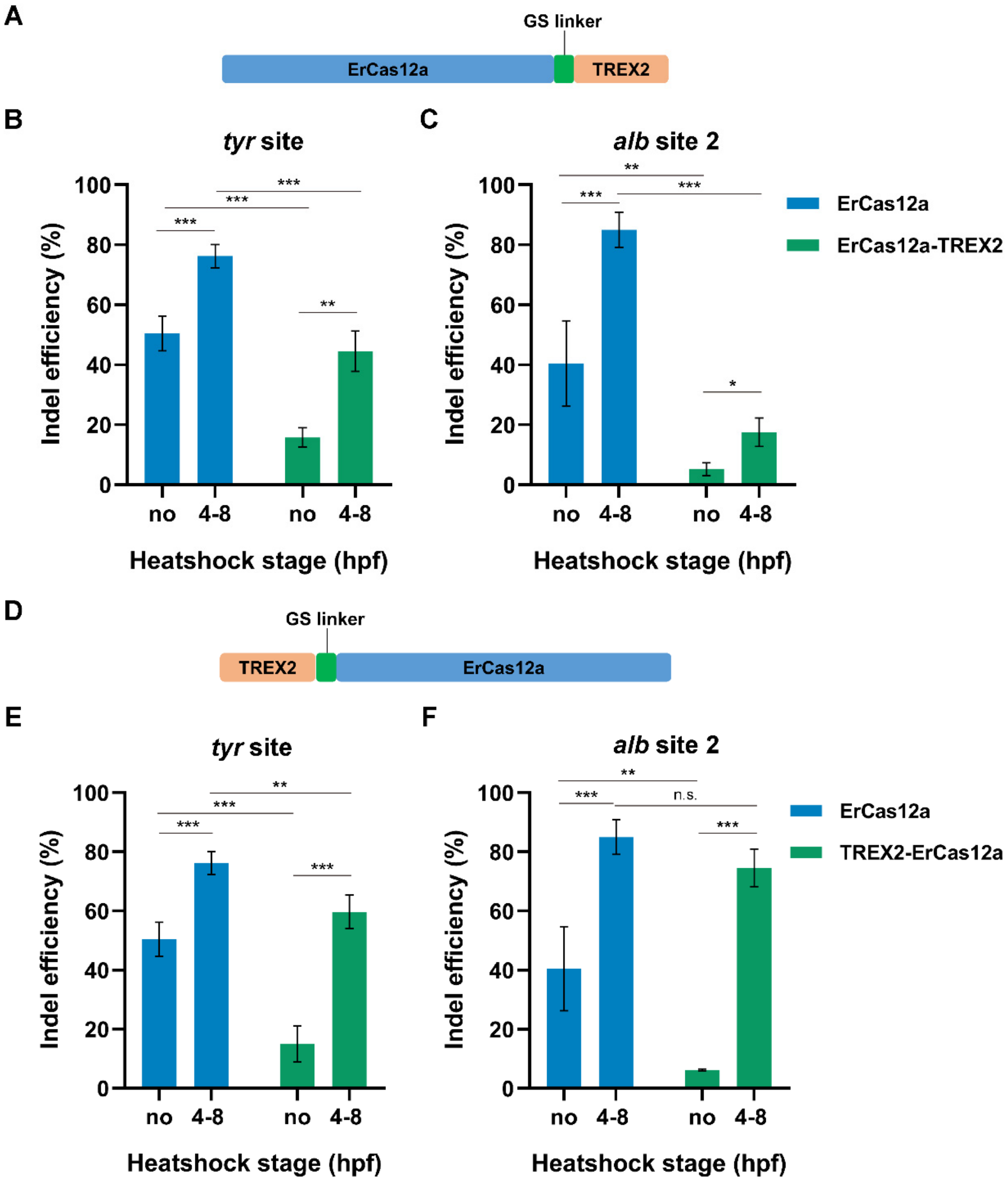

Figure S4. Mutagenesis capacity of N- or C-terminal fusion of TREX2 to ErCas12a. (**A**) Schematic diagram of ErCas12a fused with TREX2 at the C-terminus. The two proteins are separated by a GS linker. (**B**) Indel efficiency in embryos injected with *ErCas12a-TREX2* mRNA and *tyr* pre-crRNA under heatshock or non-heatshock conditions. Results were compared with *ErCas12a* mRNA injection under the same conditions. (**C**) Indel efficiency in embryos injected with *ErCas12a-TREX2* mRNA and *alb* pre-crRNA2 under heatshock or non-heatshock conditions. Results were compared with *ErCas12a* mRNA injection under the same conditions. (**D**) Schematic diagram of ErCas12a fused with TREX2 at the N-terminus. The two proteins are separated by a GS linker. (**E**) Indel efficiency in embryos injected with *TREX2-ErCas12a* mRNA and *tyr* pre-crRNA under heatshock or non-heatshock conditions. Results were compared with *ErCas12a* mRNA injection under the same conditions. (**F**) Indel efficiency in embryos injected with *TREX2-ErCas12a* mRNA and *alb* pre-crRNA2 under heatshock or non-heatshock conditions. Results were compared with *ErCas12a* mRNA injection under the

same conditions. Data represents mean +/- s.d. of at least 3 independent replicates. Unpaired two-tailed Student's *t*-test was used to calculate *p* values (* $p < 0.05$; ** $p < 0.01$; *** $p < 0.001$; "n.s." indicates the difference is not significant).

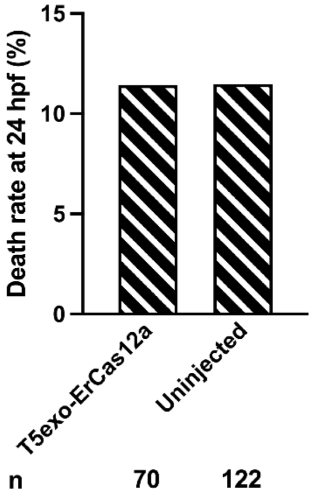

Figure S5. Evaluation of the embryo mortality after T5exo-ErCas12a system injection. Embryos were injected with *T5exo-ErCas12a* mRNA and *tyr* pre-crRNA and death rate was calculated at 24 hpf. Uninjected embryos were included as control. Number of embryos evaluated (n) is shown for each condition.

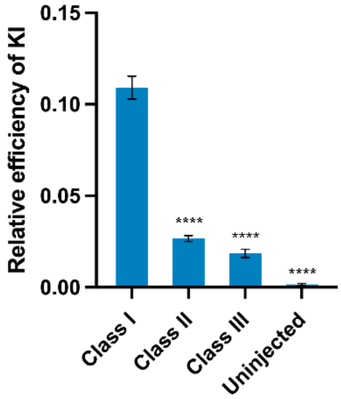

Figure S6. Evaluation of KI efficiency among different fluorescence groups mediated by T5exo-ErCas12a. Genomic qPCR results showed relative efficiency of KI donor integration among different embryonic groups strongly correlated with the classification based on the distribution of fluorescent signals. Data represents mean +/- s.d. of 3 independent replicates. Unpaired two-tailed Student's *t*-test was used to calculate *p* values (**** $p < 0.0001$) between Class I group and others.

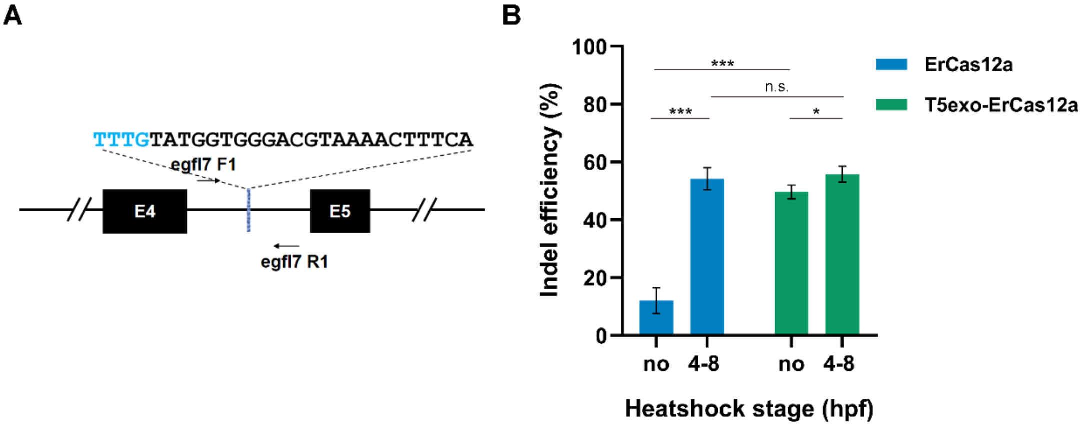

Figure S7. The effect of heatshock on T5exo-ErCas12a at the *egfl7* locus. (**A**) Schematic diagram and sequence information of the ErCas12a target site on the fourth intron of *egfl7*. The PAM sequence is indicated in blue characters. (**B**) Comparison of indel efficiency of embryos injected with *egfl7* pre-crRNA together with *ErCas12a* or *T5exo-ErCas12a* mRNA under heatshock or non-heatshock conditions. Data represents mean +/- s.d. of at least 3 independent replicates. Unpaired two-tailed Student's *t*-test was used to calculate *p* values (* $p < 0.05$; *** $p < 0.001$; "n.s." indicates the difference is not significant).

A

***tyr* site T5exo-ErCas12a TA**

Ref.	TTCCCCCGAGTCTGCACCACCCCAGAAGTCCT	
0	TTCCCCCGAGTCTGCACCACCCCAGAAGTCCT	6
-18	TTCCCCCGAGT------------------CCT	9
-17	TTCCCCCGA-----------------AGTCCT	4
-14	TTCCCCCGAGTCTG--------------TCCT	2
-19	TTCCCCCGAG-------------------CCT	1
-23	TTCC-----------------------GTCCT	1
-15	TTCCCCCGAG---------------AAGTCCT	1
-13	TTCCCCCGAGTCTG-------------GTCCT	1
-8,1	TTCCCCCGAGTCTGCA--------GAAGTCCT	1
-8,2	TTCCCCCGAGTC---A-----CCTGAAGTCCT	1
-11	TTCCCCCGAGTC--C---------GAAGTCCT	1
Total		28

B

***alb* site 2 T5exo-ErCas12a TA**

Ref.	GGAATTCTGCTACGCTGTT GAGGCTGCGTTTG	
0	GGAATTCTGCTACGCTGTT GAGGCTGCGTTTG	4
-17	GGAATTCTGC--------- --------GTTTG	4
-18	GGA---------------- --GGCTGCGTTTG	3
+1,mismatch	GGAATTCTGCGTTTGGAAGGGAGGCTGCGTTTG	3
-11	GGAATTCTG---------- -AGGCTGCGTTTG	3
-16	GGAATTCTGCTAC------ ----------TTG	2
-2	GGAATTCTGCTACGCTGT- -AGGCTGCGTTTG	2
-4,mismatch	GGAATTC--CTT--CTGTC GAGGCTGCGTTTG	2
-12	GGAAT---G------T-TT --GGCTGCGTTTG	2
-9,1	GGAATTCTGCTA------- --GGCTGCGTTTG	1
-9,2	GGAATTC--C-A------T GAGGCTGCGTTTG	1
-10	GGAATTCTG---------- GAGGCTGCGTTTG	1
-14	GGAATTCTG---------- ----CTGCGTTTG	1
-113	........--------------------- GAGGCTGCGTTTG	1
Total		30

Figure S8. Evaluation of the indel mutation forms induced by T5exo-ErCas12a. (A) Sequence alignment indicates indel mutation pattern at *tyr* site caused by injection of *T5exo-ErCas12a* mRNA and *tyr* pre-crRNA. (**B**) Sequence alignment indicates indel mutation pattern at *alb* site 2 caused by injection of *T5exo-ErCas12a* mRNA and *alb* pre-crRNA2. The indel mutation sequences in both experiments were obtained by PCR amplification of the genomic region of the target site followed by TA cloning and Sanger sequencing. The number before each sequence indicates the mutation form, while the number after the sequence indicates the total number of sequenced single clones of each particular indel form.
